# Comparison of canal transportation and centering ability of manual K-files and reciprocating files in glide path preparation: a micro-computed tomography study of constricted canals

**DOI:** 10.1186/s12903-021-01440-3

**Published:** 2021-02-23

**Authors:** Jing-Yi Liu, Zhi-Xiong Zhou, Wei-Ju Tseng, Bekir Karabucak

**Affiliations:** 1 Center of Dental Medicine, China-Japan Friendship Hospital, 2 Ying-Hua-Yuan East Street, Chaoyang District, Beijing, 100029 China; 2 Department of Pediatric Dentistry, School and Hospital of Stomatology, Peking University, 22 South Zhongguancun Avenue, Haidian District, Beijing, 100081 China; 3 Department of Endodontics, School of Dental Medicine, University of Pennsylvania, 240 S 40th St, Philadelphia, PA 19104 USA

**Keywords:** Glide path, Reciprocating motion, Optimum Glide Path (OGP), Manual motion, Micro-computed tomography (Micro-CT)

## Abstract

**Background:**

Optimum Glide Path (OGP) is a new reciprocating motion aiming to perform efficient glide path preparation in constricted canals. The aim of this study was to investigate and compare manual and OGP movement in terms of canal transportation and centering ability in glide path preparation of constricted canals.

**Methods:**

Thirty constricted mesial root canals of mandibular molars, with initial apical size no larger than ISO#8, were selected and negotiated with #6–#8 K-files under the microscope. Canals were randomly divided into two experimental groups**:** Group 1 (MAN, n = 15): Glide path was established by using #10-#15 stainless steel K-files manually; Group 2 (OGP, n = 15): #10-#15 Mechanical Glide Path super-files were used with OGP motion (OGP 90°, 300 rpm). Each instrument was used to prepare only 2 canals (as in one mesial root). Canals were scanned before and after glide path preparation with micro-computed tomography (micro-CT) to evaluate root canal transportation and centering ratio at 1, 3 and 5 mm levels from the root apex. File distortions and separations were recorded. Paired *t*-test was used to statistically evaluate the data (*P* < .05).

**Results:**

Group 2 showed a significantly lower transportation value than group 1 at 1-mm and 3-mm levels (*P* < .05), however the difference at 5-mm level was not significant. There was no significant difference regarding the centering ratio between the groups. Six #10 K-files were severely distorted in group 1, while no file separation or distortion was found in group 2.

**Conclusions:**

OGP motion performed significantly less canal transportation (apical 3 mm) and file distortion during glide path establishment in constricted canals comparing to manual motion, while the centering ability between the two was similar.

**Clinical relevance:**

OGP reciprocating motion provides a safer and efficient clinical approach compared to traditional manual motion in glide path establishment with small files in constricted canals.

## Background

The term “glide path” has been addressed in endodontics since the early 2000s, referring to a smooth tunnel pathway from the canal orifice toward the physiologic terminus [1]. Glide path establishment has been considered a crucial and initial step for safer and efficient biomechanical root canal preparation [2–4]. It could secure an open pathway to the canal terminus that subsequent instruments can follow [1]. Establishing a glide path may significantly reduce the risk of canal modifications like canal transportation, ledging, and zipping, also prevent rotary instrument separation by decreasing torsional stress to a certain extent [4–6]. In narrow, tight, and calcified canals, canal negotiation and glide path preparation may face a significant challenge; meanwhile, the previously mentioned procedural errors are more prone to occur [7]. Cvek et al. [8] not surprisingly found that irretrievable instrument separation mostly happens in constricted and calcified root canals.

Instruments like stainless steel #10–#20 K-files have been recommended to establish a glide path, with a manual watching-winding or balanced force motion [5, 6]. However, establishing a glide path with stainless steel files in such manner could be very time consuming, especially in advanced cases; also, it has many limitations and drawbacks, including an increased incidence of canal transportation [9, 10]. To overcome some of the problems with manual motion, for faster preparation and superior anatomy preservation, NiTi rotary glide path files with continuous rotation have been introduced [11, 12]. Yet in calcified and constricted canals, the screw-in effect of continuous rotation motion may cause instantaneous torsional overload, leading to file breakage [3].

A slow-speed handpiece combining alternate reciprocation and rotating reciprocation, the “Optimum Glide Path (OGP)”, was introduced by J. Morita (Osaka, Japan). The OGP kinematics mainly aims to mimic the watch-winding and balanced force motion [13]. Briefly, file motion starts with alternate movement, with a same angle followed by reciprocating movement in which the counterclockwise angle is larger than the clockwise (90°/120°, 180°/270°, and 240°/330°). Then OGP kinematics continuously alternates between the alternate and rotating reciprocation motions. OGP with the smallest angular increment has shown the highest mean time-to-fracture when compared with continuous rotation or other rotation angles, indicating a much safer use in difficult cases [14]. However, the performance of OGP motion regarding canal transportation and centering ability in constricted canals still remains unclear. Therefore, the purpose of this study was to investigate and compare manual and OGP movement in terms of canal transportation and centering ability in glide path preparation of constricted canals.

## Methods

### Sample selection

Research protocol was approved by the Research Ethics Committee (PKUSSIRB-201417105). Fifteen extracted mandibular molars with intact mesial roots and closed apices were chosen, based on the power analysis calculation in a previous study by our institution [15]. Briefly, an effect size of 1.93, an alpha-type error of 0.05 and a power beta of 0.95 were input together into an independent samples test from the *t*-test family (G*Power 3.1.9.3 for Macintosh; Heinrich Heine, Dusseldorf, Germany). The results implied a minimum sample size of 9 samples/group. Taking another canal transportation and centering ability study into consideration [16], a sample size of 15 canals/group was eventually decided. Teeth with fractures, restorations or caries were excluded. Pre-operative radiographs from different angles (a horizontal angle of 0° and a 30° from the mesial) were taken to make sure all mesial root canals were separated and narrow, with curvatures of 20° to 40° [17].

### Pre-operative micro-CT scan

The teeth were decoronated approximately 2 mm above the cemento-enamel junction (CEJ) using diamond burs.

A micro-CT system (vivaCT-40, Scanco Medical, Bassersdorf, Switzerland) at 21 μm nominal voxel size, 56 kVp energy, 142 μA intensity, and 200 ms integration time was used to acquire scans. The region of interest extending from the CEJ to the apex were analyzed and reconstructed by Scanco evaluation software (v6.6, Scanco Medical). Samples were verified that the selected canals were separate and constricted, with initial apical size not larger than #8.

### Glide path preparation

A total of 30 canals, 15 mesiobuccal (MB, n = 15) and 15 mesiolingual (ML, n = 15) canals, were selected from the sample teeth and negotiated with #6-#8 stainless steel K-files (Roydent, Johnson City, TN, USA) to achieve apical patency. The working length was determined under an operating microscope by inserting #8 K-file slightly passing the canal terminus and subtracting 0.5 mm from this measurement.

Canals were randomly assigned into two experimental groups (www.random.org) [18]. Briefly, stratification random sampling was applied, with canals being stratified as MB and ML first and followed by simple random sampling. **Group 1**: Glide path was established by using pre-curved #10-#15 stainless steel K-files (Roydent) manually to the working length; **Group 2**: Mechanical Glide Path (MGP) super-files #10-#15 (MANI Inc., Japan) were used with OGP setting (OGP 90°, 300 rpm) on Tri-Auto ZX2 cordless motor (J. Morita CORP.). Following the manufacturer’s recommendations, in-and-out pecking movement as well as horizontal reciprocating movement were carefully combined and conducted.

RC-Prep (Premier, Plymouth Meeting, PA, USA) was used as a lubricating agent and the canals were irrigated with copious 3% sodium hypochlorite. Each instrument, manual or mechanical, was used to prepare only 2 canals (as in one mesial root). Any file distortion and separation were observed under microscope and recorded in both groups, and specimens with separated files were excluded at this point. All the preparation was performed by a single dentist with expertise in both preparation techniques.

### Post-operative micro-CT scan and image analysis

A total of 30 canals (n = 15 in each group) was scanned post-operatively. To ensure the standardization of the specimens during scanning, silicon rubber discs were used as positioners to keep specimens in the same relative positions for both pre- and post-operative scans, with the same protocol and parameter settings.

**Fig. 1:**
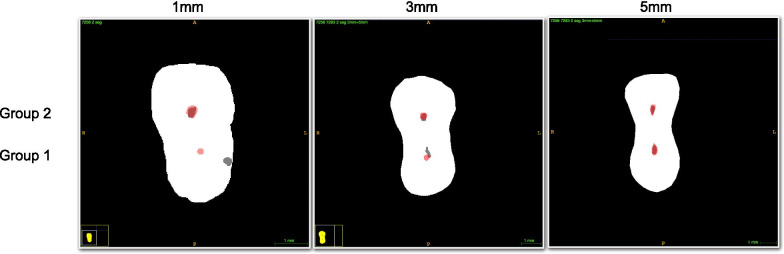
Representative images from different cross-section levels in each group, showing how before and after-operative Micro-CT scans were co-registered and overlapped. Grey indicates uninstrumented canal space, while red indicates instrumented canal space. Group 1: Glide path was established by using #10-#15 stainless steel K-files manually; Group 2: #10–#15 Mechanical Glide Path super-files were used with OGP motion (OGP 90°, 300 rpm)

3D Slicer 4.6.2 software [19] was used to co-register the pre- and post-operative scans, followed by the application of ITK-SNAP 3.6.0 software to overlap the co-registered images **(**Fig. 1). Fiji 1.46r software (ImageJ, Madison, WI) was used to do the measurements, so that canal transportation and the centering ratio were calculated at three cross-section levels that corresponded to 1-mm, 3-mm, and 5-mm distance from the root apex by using the equations described by Gambill et al. [20].

### Statistical analysis

Statistical analysis was performed with GraphPad Prism 6.2 software (GraphPad Software Inc., San Diego, CA, USA). Shapiro–Wilk normality test was applied to confirm the normality distribution of data (*P* > 0.05), followed by paired *t*-test to compare between two groups at different levels. Values of *P* less than 0.05 were considered statistically significant.

## Results

**Fig. 2:**
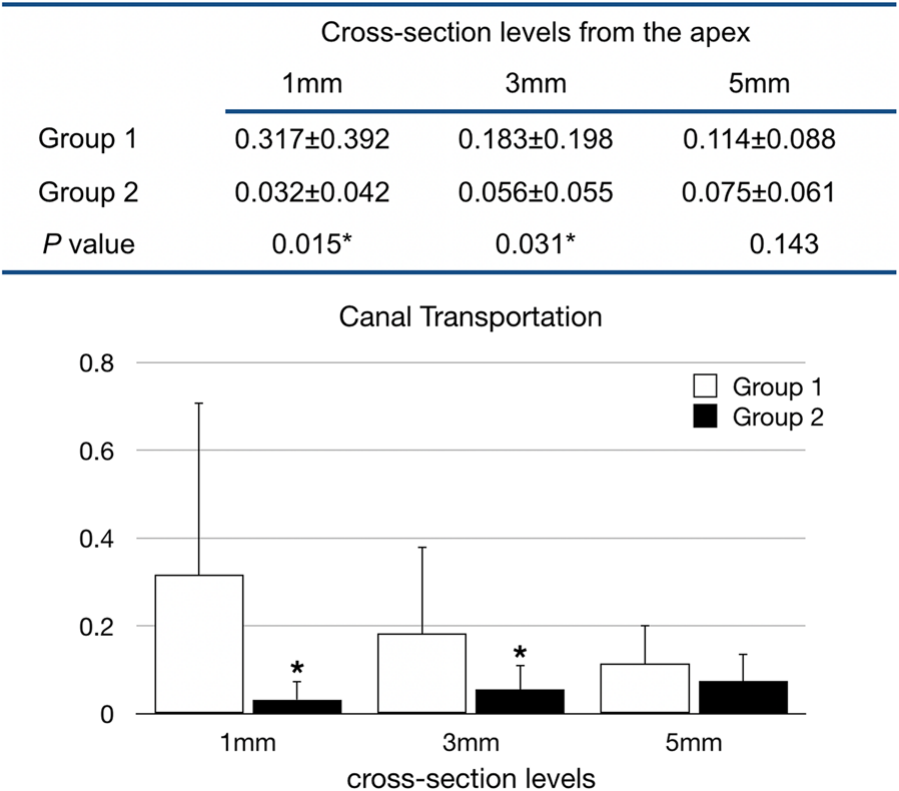
Mean canal transportation values and statistical analysis in two experimental groups (n = 15). A result of 0 indicates no canal transportation (*P < 0.05). Group 1: Glide path was established by using #10–#15 stainless steel K-files manually; Group 2: #10–#15 Mechanical Glide Path super-files were used with OGP motion (OGP 90°, 300 rpm)

**Fig. 3:**
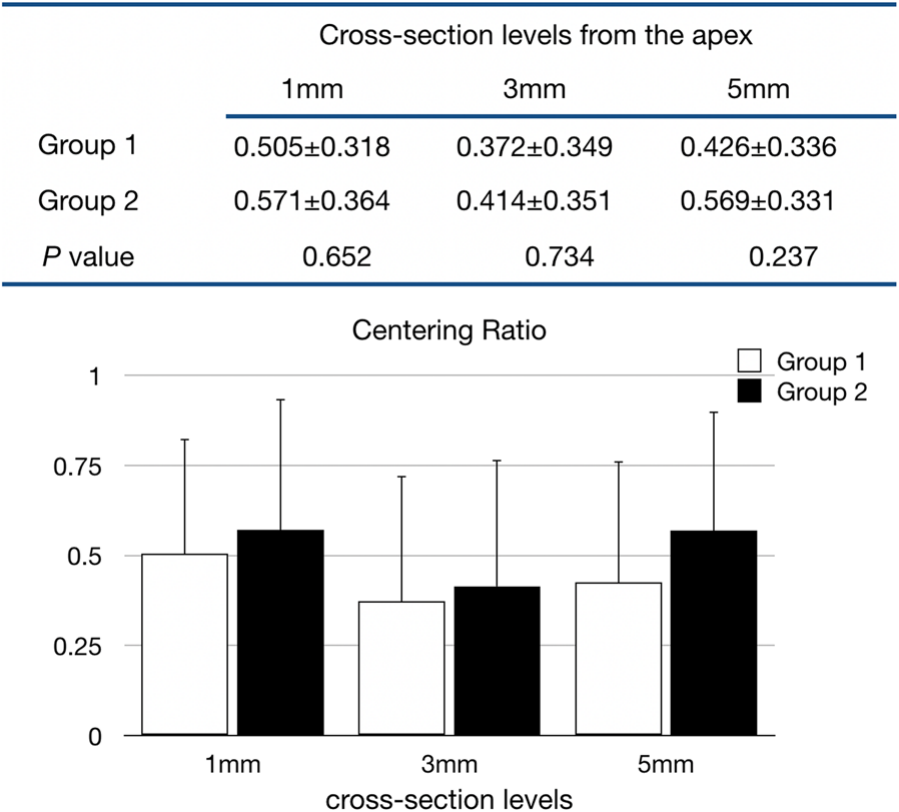
Mean canal centering ratio values and statistical analysis in two experimental groups (n = 15). A result of 1 indicates perfect centering ability. Group 1: Glide path was established by using #10-#15 stainless steel K-files manually; Group 2: #10–#15 Mechanical Glide Path super-files were used with OGP motion (OGP 90°, 300 rpm)

Nine MB and six ML canals were included in group 2, and group 1 contained six MB and nine ML canals. Group 2 showed a significant lower transportation value than group 1 at both 1-mm and 3-mm cross-section levels (*P* < 0.05), while the difference at 5-mm level was not significant (Fig. 2). Regarding to the ability of maintaining within the central axis of the root canal, the differences between the OGP reciprocating motion and manual preparation at all three cross-sections studied were not statistically significant (Fig. 3).

A total of six #10 K-files were severely distorted in group 1, at the apical 1 to 3-mm of the files; whereas no file distortion or separation was found in group 2.

## Discussion

The present study revealed that OGP motion has a significant advantage and positive impact on the preservation of apical canal anatomy during glide path establishment in constricted canals, with significant less canal transportation and file distortion.

It is essential to address the differences between a glide path establishment and maintaining apical patency, in terms of their major goals and procedures. Glide path development is an enlargement to a size approaching the subsequent rotaries’ tips, at least larger than the file’s core diameter, therefore to ensure a safe and smooth passage [1]. The concept of apical patency can be defined as ‘the repeated penetration of the apical foramen with a small file during instrumentation to prevent the accumulation of debris and leave the foramen unblocked, i.e., patent’ [21]. The patency file should be smaller than the instrument that binds to the foramen, to be effective in offering a lesser risk of extruding toxic products and dentin fragments into periapical space [21]. In most of the cases, a size #8 file taken 0.5–1 mm long to establish patency contacts the desired endpoint of the preparation with a diameter approaching the tip size of a #10 file [1].

American Association of Endodontists defines transportation as ‘removal of canal wall structure on the outside curve in the apical half of the canal due to the tendency of files to restore themselves to their original linear shape during canal preparation’ [22]. Our study showed that MGP super-files together with OGP 90° motion created significantly less canal transportation in the apical 3 mm of constricted canals than traditional K-flies. Wu et al. [23] stated that apical transportation of more than 300 μm may have a negative effect on the seal of root fillings. Canal transportation can lead to inappropriately dentin removal, with a higher risk of canal curvature modification and ledge formation. Many factors may contribute to certain degrees of canal transportation, such as motion type, canal morphology, design, and alloy of the instrument [24]. According to a new classification scheme proposed by Gambarini et al. [14], OGP is a combined motion of alternate and rotating reciprocation. Alternate reciprocation can be considered the safest motion, as it features clockwise = counterclockwise (usually less than 90°) and does not result in a full rotation. Whereas one angle of motion is larger than the other in rotating reciprocation, like Reciproc (150°/30°, Dentsply VDW, Munich, Germany) and WaveOne (170°/50°; Dentsply Maillefer) [25], and a series cycles ultimately form a full rotation inside the canal. It’s worth noting that the rotating effect given by this difference between clockwise and counterclockwise movements is important since it maintains the cutting efficiency and apical progression; moreover, this asymmetry in rotating reciprocation has the advantage of limiting the angel of rotation in the cutting verse under the endurance limit of the instrument [25]. Our finding indicates that the combination of watch-winding, and balanced force motion of OGP certainly has easier and better control during glide path preparation; whereas manual motion requires much more effort to maintain the original anatomy.

The mean centering ratio is a measure of the ability of the instrument to stay centered in the canal. A previous study revealed that reciprocating motion may promote the canal centering ability, and reduce the risk of root canal deformity [26]. Our data demonstrated similar findings, as OGP has a better centering ratio in apical 5 mm, however the results were not statistically significant.

Endodontic instrument separation may be created as a result of two distinct modes or their combination. While cyclic flexural fatigue may happen when continuous tension–compression cycles generate in curved canals overtime, torsional failure occurs when the file tip gets locked in the calcified canal; however, file shank continues to rotate [27]. Our study demonstrated a notable difference between the two experimental groups. As each instrument investigated was used to prepare only 2 canals (as in one mesial root), not a single MGP super-file under OGP 90° motion distorted or broke, while there were 6 K-files severely distorted in group 1. Several underlying mechanisms might contribute to this phenomenon. The combined alternate + rotating reciprocation movement acts to reduce the stresses of taper lock and screw-in effect, minimizes torsional and flexural stresses on the instrument, thus decreases the chance of instrument breakage [28, 29]; moreover, studies have shown that rotation angle and angular increment are related to the distribution of stress [30, 31]. OGP 90° has better cyclic fatigue and torsional resistance than OGP 180°, OGP 240°, and continuous rotation motion [14]. The up-and-down pecking motion of the handpiece with 1 mm short pecking depth may also lower the torsional stress accumulation and overload during glide path establishment since the contact time, and binding area between the instrument and dentin walls become short and small [32, 33].

Further research is still in need to evaluate other aspects of OGP motion using NiTi instruments, with comparison not just to manual motion but more importantly to continuous rotation and other single rotating reciprocation motions, so that we can have a complete understanding and clinical approach regarding the glide path establishment in constricted canals.

## Conclusion

It can be concluded that OGP reciprocating movement performed significantly less canal transportation (apical 3 mm) and file distortion during glide path establishment in constricted canals comparing to manual motion, while the centering ability between the two was similar. These favorable results of OGP movement indicate its potential application as a viable alternative to manual motion in glide path preparation of constricted canals, yet further research is needed to compare this novel motion with other rotary movements and on other parameters.

## Data Availability

The datasets used and analyzed during the current study are available from the corresponding author on reasonable request.

## Abbreviations

OGP: Optimum glide path
CEJ: Cemento-enamel junction
MGP: Mechanical glide path
